# Clonally stable Vκ allelic choice instructs Igκ repertoire

**DOI:** 10.1038/ncomms15575

**Published:** 2017-05-30

**Authors:** Rena Levin-Klein, Shira Fraenkel, Michal Lichtenstein, Louise S. Matheson, Osnat Bartok, Yuval Nevo, Sebastian Kadener, Anne E. Corcoran, Howard Cedar, Yehudit Bergman

**Affiliations:** 1 Department of Developmental Biology and Cancer Research, Institute for Medical Research Israel–Canada, Hebrew University Medical School, Jerusalem 91120, Israel; 2 Nuclear Dynamics Programme, Babraham Institute, Babraham Research Campus, Cambridge CB22 3AT, UK; 3 Biological Chemistry Department, Silberman Institute of Life Sciences, Edmund J. Safra Campus, The Hebrew University, Jerusalem 91904, Israel; 4 Computation Center, Hebrew University–Hadassah Medical School, Jerusalem 91120, Israel

## Abstract

Although much has been done to understand how rearrangement of the Igκ locus is regulated during B-cell development, little is known about the way the variable (V) segments themselves are selected. Here we show, using B6/Cast hybrid pre-B-cell clones, that a limited number of V segments on each allele is stochastically activated as characterized by the appearance of non-coding RNA and histone modifications. The activation states are clonally distinct, stable across cell division and developmentally important in directing the Ig repertoire upon differentiation. Using a new approach of allelic ATAC-seq, we demonstrate that the Igκ V alleles have differential chromatin accessibility, which may serve as the underlying basis of clonal maintenance at this locus, as well as other instances of monoallelic expression throughout the genome. These findings highlight a new level of immune system regulation that optimizes gene diversity.

## Abstract (web summary)

B cell development involves sequential rearrangement of the immunoglobulin chains, but fine control over the selection process remains a mystery. Here the authors show that individual alleles in pre-B cells are clonally unique and result from stochastic activation of V gene segments to induce optimal generation of a diverse repertoire.

Rearrangement of immune receptor loci in B and T lymphocytes takes place in an ordered developmental manner using transcription factors and regulatory elements to open up and turn on the rearrangement process at each individual cluster during its specific stage of differentiation^1^,^2^,^3^,^4^,^5^. In the B-cell lineage, the IgH locus is activated first in pro-B cells, whereas the Igκ region gets turned on and rearranged only at a later stage of development in the small pre-B-cell compartment. This activation occurs initially on only one allele, which undergoes J–C region demethylation and proceeds with rearrangement^6^,^7^,^8^ seemingly choosing from the full range of V segments^9^.

Originally, it was thought that at the time of rearrangement the two κ alleles in each cell are equal substrates for activation, with the choice being made in a stochastic manner^10^,^11^. Previous work in our laboratory, however, has indicated that this is probably not the case and the decision is actually of an instructive nature, with the two alleles first becoming marked by asynchronous replication at the early lymphoid progenitor stage followed later by opening of the κ J–C region specifically on the early allele. Through the use of pre-B-cell clones, it was then demonstrated that it is this same allele that undergoes the first rearrangement in each cell^12^. The κ locus is distributed over a large 3 Mb region carrying ∼140 different V segments^13^ and this domain already has an accessible chromatin conformation at the pre-B-cell stage even prior to the initiation of rearrangement^14^,^15^,^16^. However, the actual chromatin structure and transcription pattern of individual V segments on the two alleles has not yet been identified.

In this study, we use hybrid C57BL/6/Castaneous (*B6/Cast*) pre-B-cell clones to examine the chromatin and transcriptional state of the κ locus V segments in an allele-specific manner. The results indicate that each parental chromosome independently activates a select number of V segments. Once chosen, these activity states are then maintained in clonal populations probably through their highly stable accessible chromatin structure. In the case of the Igκ locus, this ‘choice' of V segments seems to generate alternate recombination patterns on each allele, thus providing a mechanism for enhancing the chances of each B cell to produce functional antibodies. Furthermore, this same chromatin-based model may also serve as the basis for the maintenance of differential expression at a large number of monoallelic loci present in the genome.

## Results

### V region allele specific histone modification states

**Figure 1:**
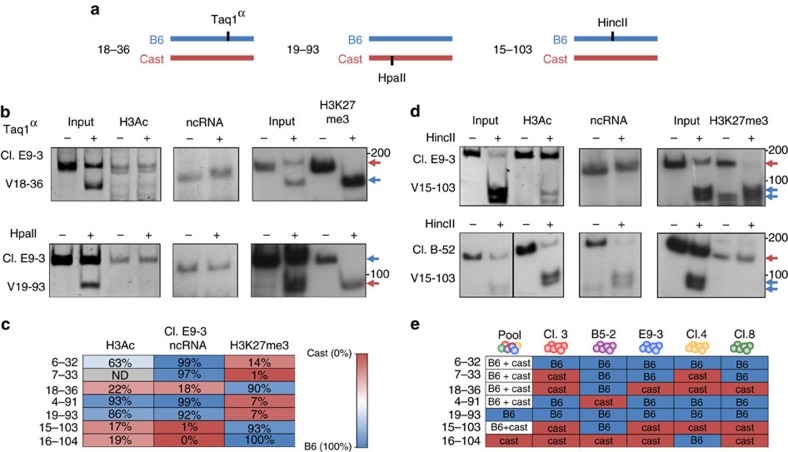
Vκ segments are monoallelically marked in pre-B cells. (**a**) Schematic view of Vκ segments from *B6/Cast* mice. Allele-specific restriction sites generated by single-nucleotide polymorphisms (SNPs) between the two alleles are marked in black. (**b**) Sample restriction analysis gels from 2 different Vκ segments performed on ChIP-enriched DNA and RNA from clone E9-3. Expected positions of Cast and B6 alleles following restriction is marked with red and blue arrows, respectively. (**c**) Percent of the B6 allele within the ChIP bound fraction/cDNA in clone E9-3, as quantified from the fraction of the PCR product cut in comparison to input, where the two alleles are present in equal proportions. Red to blue heatmap indicates Cast to B6 levels. Quantifications were done using EZquant software. (**d**) Sample restriction analysis gels from the same Vκ segment (15-103) performed on ChIP-enriched DNA and RNA from clones E9-3 and B-52. Expected positions of Cast and B6 alleles following restriction is marked with red and blue arrows, respectively. (**e**) Summary of active alleles across 5 different pre-B cell clones and pools of bone-marrow-derived pre-B cells as determined by H3ac enrichment, ncRNA transcription and lack of H3K27me3 enrichment.

To determine the pattern of V region activation states, we analysed histone acetylation over select V segments in pre-B-cell clones derived from chimeric *B6/Cast* mice. Since, in general, the sequences of the two alleles differ by about 1% genome wide^17^, we were able to identify many polymorphic sites that could be used to determine the histone acetylation pattern of each allele separately. We first chose a single clone (E9-3) and carried out anti-histone H3Ac ChIP, which was then assayed by PCR analysis of various V segments within the κ locus, using polymorphisms at restriction-enzyme binding sites to distinguish between the alleles (Fig. 1a). In a striking manner, it appears that individual Vs are acetylated in a monoallelic manner. Thus, for example, *V*_18–36_ was found to be packaged with acetylated histones on the Cast allele, while being relatively unacetylated on the B6 allele (Fig. 1b). Conversely, ChIP analysis using an antibody against H3K27me3, a signature of closed chromatin, showed heavily skewed enrichment on the unacetylated B6 allele. These structural measurements are consistent with non-coding RNA (ncRNA) analysis indicating that only the acetylated allele is being transcribed. For a different segment (*V*_19–93_) in the same E9-3 pre-B-cell clone it was the B6 allele that had an acetylated histone pattern accompanied by preferential transcription. In contrast, the Cast allele was not only found to be relatively unacetylated, but even carried the repressive H3K27me3 mark. Using a similar approach, we found that additional Vs also demonstrated allele-differential ‘opening' and this was always in correlation with ncRNA transcription (Fig. 1c). The transcribed alleles were also found to be marked with H3K4me1 and H3K4me2, additional modifications associated with active chromatin (Supplementary Fig. 1).

These results indicate that on any given allele not all the Vs are chosen to be activated and this decision then appears to be maintained in a clonal manner in the E9-3 line used for these studies. In order to investigate whether these choices are possibly of genetic origin, simply reflecting sequence differences between the Cast and B6 genomes, we next carried out this same analysis on a number of different clonal pre-B-cell lines (Fig. 1d,e). This experiment revealed that each clone actually has a different pattern of V region availability independent of allele origin. As an example, *V*_15–103_ is open and expressed almost exclusively on the Cast allele in E9-3, while in clone B-52, it is the B6 allele that is preferentially chosen. A summary of seven different V segments in various independent cell clones indeed confirmed that each one has its own unique pattern of allelic choice with the open (acetylated) copy being transcribed in pre-B cells. As expected, bone-marrow pools from which all pre-B-cell clones are derived show biallelic expression of almost all V regions, probably because they represent a mixture of individual clones that choose the V segment on each allele in a stochastic manner. Nonetheless, some V segments were found to display a strong genetic bias for one particular allele (Fig. 1e).

### V region allele-specific transcription

**Figure 2:**
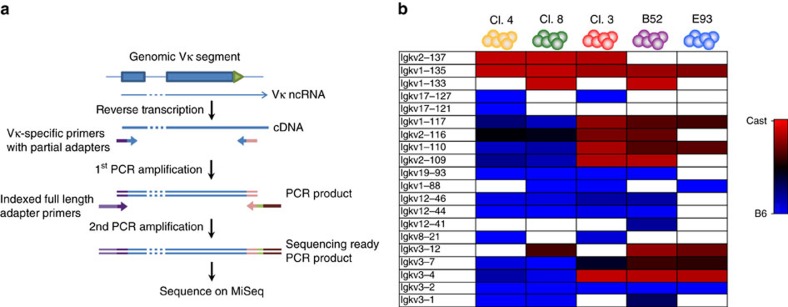
Amplicon sequencing of Vκ ncRNA. (**a**) Overview of Vκ amplicon sequencing workflow on a generic Vκ segment. (**b**) Heatmap of B6/Cast ratios of RNA expression for 20 different Vκ segments in pre-B-cell clones, as assayed by amplicon sequencing. Red to blue heatmap indicates linear Cast to B6 levels on a scale from 0 (100% Cast) to 1 (100% B6). White indicates that the Vκ segment was not amplified.

To obtain an expanded picture of V region choice, we developed a multiplex PCR method to assay ncRNA transcription for a small repertoire of 20 different Vs (Fig. 2a). In any given clone, we found that each V was either silenced on both alleles, expressed preferentially on one allele (B6 or Cast) or displayed a biallelic pattern (Fig. 2b). This suggests that the original choice of Vs in each clonal population may be based on a stochastic process. Strikingly, no two clones had the exact same transcription pattern of the V segments.

**Figure 3:**
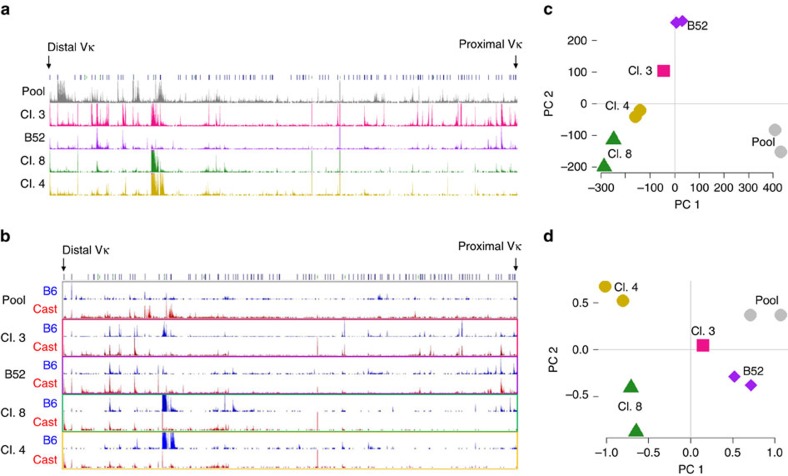
Clonally stable pre-B cell Vκ region transcription profiles. (**a**) Nuclear RNA profile over the entire Vκ region. Genomic interval and tracks displayed on UCSC genome browser, mouse build mm10. (**b**) Allele-specific nuclear RNA profile over the entire Vκ region. (**c**) Principal component analysis of normalized total ncRNA transcript profiles across all Vκ segments in biological duplicates of different pre-B-cell clones and *ex-vivo B6/Cast* bone marrow pre-B cells (pool). (**d**) Principal component analysis of allelic ratio of ncRNA transcription across all Vκ segments in biological duplicates of different pre-B-cell clones and *ex-vivo B6/Cast* bone marrow pre-B cells (pool).

As a further proof of this principle, we next adapted a method for genome-wide analysis of the total nuclear RNA fraction^18^,^19^ and quantified RNA levels over the entire V region of both alleles (Fig. 3). The overall pattern of transcription across the locus was distinct in each of the clones analysed (Fig. 3a, Supplementary Fig. 2a) and in every case, we also observed striking differences between the two parental alleles (Fig. 3b, Supplementary Fig. 2b,c), generating a kaleidoscope of gene expression (Supplementary Fig. 3). Furthermore, these profiles remain stable across multiple divisions and cell passages as confirmed by principal component analysis (PCA) (Fig. 3c,d). Interestingly, clone 3 apparently underwent deletion of the Vκ locus Cast allele in late passages. Nonetheless, the pattern of V segment expression on the B6 allele remained constant (Supplementary Fig. 4). This suggests that each allele profile is maintained independently, with no crosstalk between the alleles being required.

### V region allele-specific chromatin accessibility

**Figure 4:**
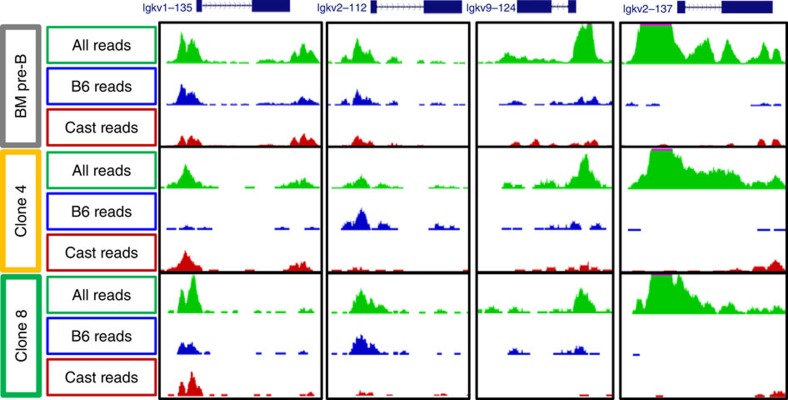
Allelic regions of Vκ promotor chromatin accessibility. ATAC-seq profiles of chromatin accessibility in pre-B-cell clones and *ex-vivo* sorted BM pre-B cells at representative Vκ segments *V*_1–135_, *V*_9–134_, *V*_2–112_ and *V*_2–137_. Total reads, regardless of allelic origin are marked in green, reads identified as originating from the B6 allele based on SNPs are marked in blue, reads identified as originating from the Cast allele based on SNPs are marked in red. It is not possible to identify the allelic source of all mapped reads, as exemplified by *V*_2–137_. Genomic intervals and tracks displayed on UCSC genome browser, mouse build mm10.

Since expression is always correlated with local histone acetylation on an allelic basis, it appears that, in general, some V segments are relatively open and accessible within the nucleus while others are in a more closed configuration (Fig. 1). To test this idea, we carried out genome-wide ATAC-seq analysis on each of the different pre-B-cell clones. In this assay, a transposase is used to insert a marker sequence into multiple sites in the genome which are then detected through sequencing analysis, with the degree of integration being proportional to the level of accessibility at each individual locus^20^. By restricting our analysis to sequences located exclusively in the Vκ region, we were able to construct a local allelic ‘openness' map of specific V segments (Fig. 4). *V*_1–135_, for example, was shown to be accessible on both the Cast and B6 alleles in a pool of pre-B cells. In clone 4, however, only the Cast allele was open, while the B6 allele showed a relatively small number of sequence reads indicating that the Cast allele is in a more accessible configuration, consistent with the finding that this V is preferentially transcribed on the same allele. In contrast, *V*_2–112_ is more open on the B6 allele in this same clone, while *V*_9–124_ is accessible on both alleles in equal measure. In clone 8, however, the pattern of chromatin accessibility is distinctly different, with both *V*_9–124_ and *V*_2–112_ being accessible on the B6 allele, while V_1–135_ is accessible on both alleles, with a skew towards Cast. Analysis of other pre-B-cell lines indicated that this allelic pattern is different in each individual clone (Supplementary Fig. 5). It should be noted that while this assay is quite robust, allowing one to identify a large number of accessible Vs, we were not always able to determine their allelic distribution, mainly because the relevant marker sequences were often too small to include nearby polymorphisms (see example *V*_2–137_ (Fig. 4)).

### Allele-specific Igκ rearrangement repertoires

**Figure 5:**
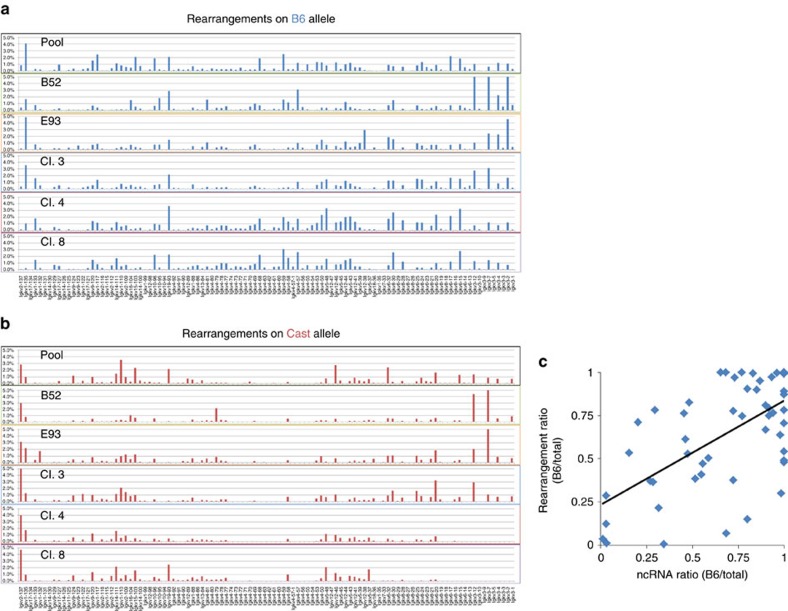
Allelic Igκ rearrangement repertoire in pre-B cells. Rearrangement repertoires of differentiated pre-B-cell clones and a pre-B-cell pool from *B6/Cast* mice were generated. Normalized percent contribution of each Vκ segment to the repertoire is presented for the (**a**) B6 and (**b**) Cast allele separately. It should be noted that in both clone 3 and clone 4 it is the B6 allele that replicates early, consistent with the observation that there are more observed recombination events on this allele. (**c**) Correlation between the allelic ratio of Vκ-segment ncRNA in pre-B cells prior to rearrangement and allelic ratio of rearranged Vκ segments following differentiation. Pearson correlation coefficient 0.62. *P*<10^−5^.

Having shown at the global level that each clone maintains a different V region accessibility and expression pattern, we then asked whether this may serve as the basis for determining the rearrangement patterns that take place following differentiation to B cells. To this end, we developed a technique to pick up RNA from rearranged molecules by using a Cκ primer together with 3′ ligation-mediated PCR and then quantitatively assayed all detectable rearrangements by large-scale sequencing (Supplementary Fig. 6). This data yielded a clonal recombination map for each allele independently (Fig. 5a,b), and with each individual clone producing a different pattern of rearrangement. Furthermore, by comparing the ratios between the B6 and Cast alleles at each V segment, we found that the degree of rearrangement following differentiation is directly correlated with the amount of ncRNA transcription for each V segment in pre-B-cell clones (Fig. 5c). It thus appears that the choice of V segment ‘opening' ultimately affects the pattern of rearrangement on each allele.

The data we have presented suggest that while the choice of Vs on each allele is mainly stochastic, some sites appear to have a fixed bias for either B6 or Cast, suggesting that genetic factors may also play a role in this choosing process. To address this question in a general manner, we carried out PCA on the ncRNA spectrum measured in several different pre-B-cell populations (Fig. 3), Strikingly, we found that all the individual clones have ncRNA patterns far removed from the profile observed in a pool of pre-B cells derived from bone marrow. This provides a good indication that genetic background only plays a relatively minor role in V region activation choice. The same idea appears to be true for the rearrangement process, as well, as careful analysis of the results in Fig. 5 demonstrates that less than 35% of the Vs are skewed (>80%) for one allele or the other in the pool.

The data in Fig. 5 demonstrate that following 48 h of induction, each clone appears to have undergone rearrangement on both alleles. To understand how this comes about, we followed the kinetics of this process for one individual clone (clone 4). Previous studies in our laboratory have already shown that the two alleles in pre-B cells are differentially marked by replication timing and J-Cκ accessibility, and that it is always the early allele which is preferentially rearranged in the initial recombination step^12^,^21^. In keeping with this, analysis after only 12 h of induction indicated that the vast majority of rearrangement events occurred on the B6 allele, which is indeed early replicating in this particular clone. As the induction process proceeds *in vitro* (in the absence of receptor feedback inhibition to terminate secondary rearrangements and selection), more cells begin to rearrange the second allele (Cast), allowing V–J recombination of brand new and different V segments (Supplementary Fig. 7).

### Global allele-specific transcription

These experiments present an intriguing picture of the Vκ region whereby individual gene segments are able to maintain an allelic pattern of chromatin accessibility in a clonal manner and in this way support stable monoallelic expression profiles. On the basis of previous studies showing that a large number of genomic loci are expressed monoallelically in differentiated ES cells^22^,^23^, we next asked whether this may also be true for the pre-B cells used in this study. Indeed, global analysis of RNA-seq data from different passages of the pre-B-cell clones used in this study indicates that the allelic transcription pattern is clonally stable and distinct from the other clones (Supplementary Fig. 8a,b). Each clone has between 2,000 and 4,000 genes, which are expressed in a monoallelic manner, consistent with the reports from other cell types^22^,^23^,^24^. Strikingly, while some genes are monoallelic in all clones analysed, others are monoallelic in only some clones, and biallelically transcribed in others, giving each clone a unique set of monoallelically expressed genes (Supplementary Fig. 8c,d). These genes had extremely diverse functions. Gene ontology analysis on the subset of genes monoallelically transcribed in all clones demonstrated an enrichment of glycoproteins (*P*=2.4 × 10^−7^), as well as proteins involved in signal transduction (*P*=9.3 × 10^−9^). These results suggest that the V-segment allelic choice may actually be part of a much wider phenomenon involving many other regions of the genome.

### Global allele-specific chromatin accessibility

**Figure 6:**
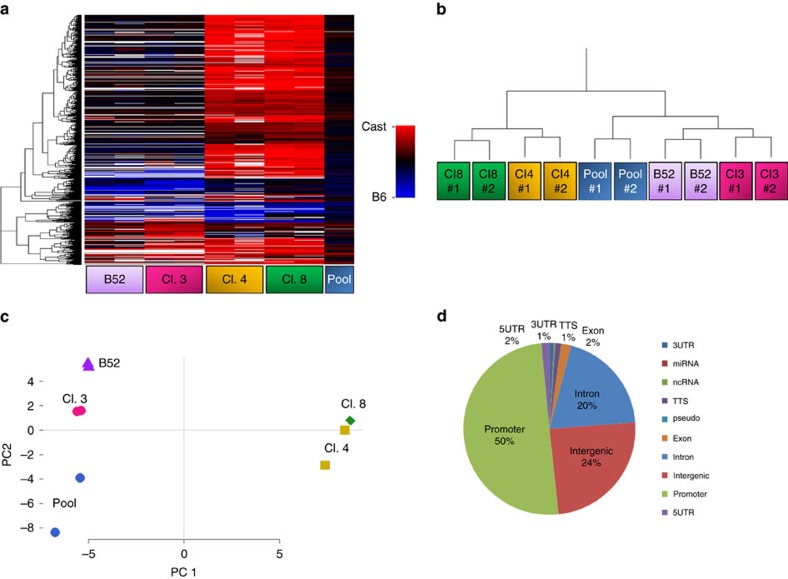
Monoallelic accessible chromatin is clonally stable. (**a**) Heatmap of allelic ratio for ATAC-seq peaks genome wide that were biallelic in pool of cells and monoallelic in at least one pre-B-cell clone. Red to blue heatmap indicates linear Cast to B6 levels on a scale from 0 (100% Cast) to 1 (100% B6). White indicates that the peak had <10 reads with allelic resolution. Hierarchical clustering (**b**) and principal component analysis (**c**) of B6/Cast ratios over ATAC-seq peaks in biological duplicates of four different pre-B-cell clones and a pool of pre-B cells derived from the bone marrow. (**d**) Genomic annotation of the relative location of accessible chromatin peaks identified in **a**.

Given clonal stability, it seemed likely that the basis for allelic differences may lie in the ability to maintain fixed alternate chromatin structures over many cell generations. To test this idea, we analysed our genome-wide ATAC-seq data from these same *B6/Cast* clones, taking advantage of multiple polymorphisms to distinguish between the two alleles. Strikingly, analysis of all of the accessible sites in the genome showed that there are indeed thousands of loci that show differential availability in individual clones even though they appear to be accessible on both alleles in a pool of pre-B cells (Fig. 6a). Furthermore, the allelic accessibility profile was found to be stable and distinct in each clone, as verified by hierarchical clustering and PCA (Fig. 6b,c).

**Figure 7:**
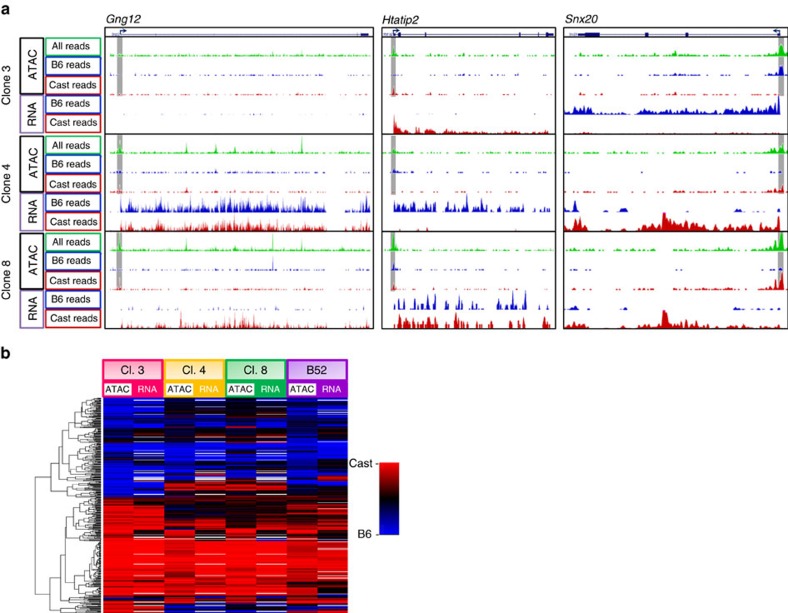
Promoter accessibility instructs allelic transcription. (**a**) ATAC-seq profiles of chromatin accessibility and allelic RNA transcription of representative genes *Gng12* (a pro-inflammatory G protein γ subunit), *Htatip2* (a pro-apoptotic protein) and *Snx20* (an endosome membrane trafficking protein) in pre-B-cell clones. Location of the gene promoter is marked in grey. Total reads, regardless of allelic origin are marked in green, reads identified as originating from the B6 allele based on SNPs are marked in blue, reads identified as originating from the Cast allele based on SNPs are marked in red. (**b**) Heatmap for allelic ratio of ATAC-seq peaks at promoters and the allelic ratio of transcription in four different pre-B-cell clones. The values are the averages of biological duplicates. Red to blue heatmap indicates linear Cast to B6 levels. Red to blue heatmap indicates linear Cast to B6 levels on a scale from 0 (100% Cast) to 1 (100% B6).

Mapping the locations of these monoallelically accessible sites showed that a large percentage of them are actually located in gene promoters (Fig. 6d). Furthermore, RNA analysis indicated that there is a strong correlation between open chromatin and transcription on an allelic basis (*χ*^2^ test *P* value <10^−15^ for each clone individually) (Fig. 7). For example, the promoter of *Gng12* is inaccessible and not expressed in clone 3, is accessible and expressed on both alleles in clone 4 and is accessible and transcribed only on the Cast allele in clone 8 (Fig. 7a). Similar correlations can be seen for other specific genes, such as *Htatip2* and *Snx20* (Fig. 7a). At the global level, as well (Fig. 7b), allele-specific promoter accessibility accurately predicts the transcription state for each allele in every clone. It thus seems likely that promoter chromatin accessibility may represent an underlying mechanism for inheritable allelic gene transcription, and this has been confirmed by other studies, as well^25^.

## Discussion

Following a programmed series of recombination events at the heavy chain locus, B cells then carry out rearrangement of the light chain region. This is done in an ordered manner with one allele being chosen in pre-B cells to undergo histone acetylation over the J–C region^8^. It is then this same allele that undergoes rearrangement by recombining with upstream V segments on the same allele^12^, but little was known about how they are chosen. Indeed, it has always been assumed that this reaction occurs in a stochastic manner with all of the V segments equally available for recombination. In this paper, we have used *Cast/B6* hybrid mice and taken advantage of genetic differences to study each allele separately. We demonstrate that in pre-B cells, the V region on both alleles undergoes a process of opening which is reflected in ncRNA transcription, histone acetylation and increased chromatin accessibility, but only a portion of the segments become activated, with the other Vs remaining relatively closed and less capable of recombination. This represents a new level of immune receptor regulation.

Our analysis suggests that in each clone, only about 30–40% of the Vs are actually activated and by examining the distribution of these sites, it appears that this is established through a stochastic process with each allele representing an independent substrate. As a result, in any one clone some Vs are activated on both alleles, some on only one allele, either B6 or Cast and the rest remain closed on both alleles. The distribution we observe is, in general, consistent with a mathematical model in which the Vs on each allele have a relatively fixed probability of activation, but these choices are also influenced by sequence biases built into the B6 and Cast genomes and may be affected by additional epigenetic markers such as replication timing, which differ between the two alleles in each clone.

Many other monoallelic genes throughout the genome also appear to be activated in a similar manner. This stochastic, as opposed to instructive system of choice, is presumably based on the idea that the factors needed to turn on these genes are present in limited quantities which are insufficient for interaction with all the targets present within the nucleus^11^,^26^. This type of mechanism is usually associated with single-gene sequences, but several examples of choice within clusters, such as those containing NK receptors^27^,^28^ or cytokine genes^29^, may also be subject to kinetics similar to that of the Vκ locus.

The most striking aspect of allelic choice seen in the Vκ region is that these patterns are preserved in a clonal manner. This implies that the initial factor-directed activation of V segments probably takes place at an earlier stage just prior to the formation of pre-B clones, which are then no longer capable of choosing but continue to maintain the initial decision of allele specificity through each cell division. It is likely that this is carried out through the formation of some sort of chromatin structure, perhaps by means of autonomously maintainable histone modification^30^,^31^,^32^,^33^. Consistent with this idea, we have demonstrated that individual V segments are either ‘open' or ‘closed' as determined by their histone modification pattern and degree of accessibility (ATAC).

By examining pre-B cells from interspecific *B6/Cast* hybrid mice we have discovered a new level of regulation within the κ light chain locus that serves to set up this region in a manner that directs the subsequent pattern of recombination. This mechanism may actually play a role in ensuring that B cells maximize their ability to produce useful antibodies. Prior to rearrangement, a subset of V segments are opened on both alleles. Initially, only the early replicating allele activates the process of recombination, which can then be carried out with any of the Vs that have already been opened. If this results in a productive antibody, the pre-B cell has completed its task and no longer attempts further recombination. In the event that no productive antibody is generated, this same allele may undergo editing by switching to a more distal available Vκ and to a different J segment. If this also fails, the same B cell attempts to carry out recombination on the second late-replicating allele (Supplementary Fig. 7)^34^. Because of the stochastic nature built into the mechanism for opening V segments, this second allele only has a limited choice of V segment partners and most of these are different than those that were activated on the first-chosen early allele. For this reason, the second allele only has a small probability of recombining with the same Vs that have already proven to be incompatible, and will mostly partner with a different set of Vs, thereby increasing its chances of generating a productive antibody. Thus, the way in which V segments are initially opened contributes to the robustness of the B cell's immune potential.

A number of different studies have documented the existence of many genes that are expressed monoallelically in a variety of different cell types, with a pattern consistent with the idea that they are activated in a stochastic manner by suboptimal concentrations of key factors^22^,^23^,^24^,^35^. We have added a new dimension to this picture by showing that these same genes have a differential chromatin structure, with one allele being more accessible than the other. It is likely that monoallelic expression represents a readout of this stable chromatin structure, perhaps explaining how these genes retain their monoallelic pattern in a clonal manner. As in the immune system, this mechanism appears to be geared at optimizing diversity to allow the expression of different alleles in each cell. The identity and functionality of the monoallelically-regulated genes vary greatly in different clones, and this probably contributes to the distinct cellular responsiveness of each cell. This may allow the immune system to effectively and robustly contend with the myriad of challenges that the organism is bound to encounter over its lifespan.

## Methods

### Animals and cells

C57BL/6 (B6) mice (Harlan) were crossed with wild-type M. castaneous (Cast) mice (Jackson Laboratories) to generate *B6/Cast* hybrid mice. Mice were housed and cared for under specific pathogen-free conditions, and all animal procedures were approved by the Animal Care and Use Committee of the Hebrew University of Jerusalem.

Cells isolated from the bone marrow of F1 female *B6/Cast* mice (8–12 weeks) were grown in RPMI 1640 media (Gibco) supplemented with 10% fetal bovine serum (Hyclone), penicillin–streptomycin (Gibco), L-glutamine (Gibco) and 50 μM of β-Mercaptoethanol (Gibco) on irradiated ST2 feeder cells. IL-7 was added to conditioned medium collected from J558L-IL7 secreting cells (as provided by A. Rolink) to select for pre-B cell populations. After 10–14 days of IL-7-mediated positive selection, cells were plated on 96-well plates in limiting dilutions to generate single-cell-derived pre-B-cell clones. Igκ locus rearrangement was induced by removal of IL-7 from the culture media for 48 h.

### Chromatin immunoprecipitation

Chromatin immunoprecipitation was performed as described previously^36^. Briefly, cells were fixated in formaldehyde and resuspended in RIPA buffer and the resulting chromatin was sonicated with a water bath sonicator to sizes ranging from 300 to 800 bp, incubated overnight with the specified antibody at 4 °C (H3Ac Millipore 06-599, H3K27me3 Millipore 07-449, H3K4me1abcam ab8895, H3K4me2 abcam ab3254) and then incubated for 3 h with protein A agarose beads (Millipore). The beads were then washed repeatedly with RIPA buffer supplemented with increasing levels of NaCl and bound DNA then released and de-crosslinked by proteinase K digestion at 65 °C for 4-15 h. DNA was purified by phenol–chloroform extraction and the quality of ChIP enrichment quantified by real-time PCR on selected V segments, relative to the input fraction. V segments were analysed for allelic bias by PCR amplification followed by allele-specific restriction enzymes (Supplementary Table 1) or Sanger sequencing after TA cloning, with the ratio of the input being used as a control.

### Allelic non-coding RNA analysis

RNA was extracted from IL-7 dependent pre-B cells, treated with DNase (Ambion) for 1 h to remove traces of genomic DNA and cDNA then prepared with the qScript RT kit (Quanta), with Vκ segments being amplified using specific primers spanning the RSS sequence to ensure it had not undergone rearrangement. PCR products were cut with allele-specific restriction enzymes, and visualized on 8% polyacrylamide TBE gels. Allelic ratios were computed based on band strength, with genomic DNA being used as a biallelic control.

For amplicon sequencing, 10 semi-degenerate primer pairs specific for a number of Vκ segment families were used to amplify 20 different Vκ segments (Supplementary Table 2). The forward primer was located within the first exon of the Vκ segment (leader sequence), while the reverse primer targeted the area immediately downstream of the second exon (RSS sequence), giving rise to an expected PCR product ∼400 bp long. The primers had the following sequences added to the beginning of the oligo: F 5′-GAGTTCTACAGTCCGACGATC-3′, R 5′-CCTTGGCACCCGAGAATTCCA-3′, corresponding to the Illumina small-RNA adapters. Following PCR amplification, excess primer was removed using Ampure XT bead size selection (Beckman-Coulter). The resulting fragments were subject to a second round of PCR, adding full length Illumina small RNA adapters. (F 5′-AATGATACGGCGACCACCGAGATCTACACGTTCAGAGTTCTACAGTCCGACGA-3′, R 5′-CAAGCAGAAGACGGCATACGAGAT-[NNNNNN]-GTGACTGGAGTTCCTTGGCACCCGAGAATTCCA-3′) with the N nucleotides signifying a six base index sequence used to differentially mark samples for demultiplexing. After the addition of the adapters, PCR fragments of the correct size (∼500 bp) that do not contain the Vκ intron were purified from a 1.5% agarose gel. Amplicon libraries were analysed by 250 bp paired-end sequencing on a Miseq instrument (Illumina). Resulting sequences were quality trimmed and aligned to a hybrid B6/Cast genome assembly using bowtie2 (ref. 37), with Cast polymorphic site substitutions based on the Sanger Mouse Genome Project database. Reads aligning to Vκ segments were then counted with HTseq-count^38^ and allelic ratios were calculated for Vκ fragments with minimum depth of 20 reads. Read depth varied from 0 to 45,000 reads for any given Vκ segment.

Stranded nuclear RNA-seq was carried out as follows. Sixteen million cells (cultured pre-B-cell clones: clones 4 and 8 in biological replicates from different cells in the same passage; Clones B52 and 3 in biological replicates from different passages) or 6 million cells (freshly sorted bone marrow B220^+^IgM^−^Cd43^−^Cd25^+^ pre-B cells pooled from five to six female 8–12-week-old B6/Cast mice, in biological duplicate) were washed with PBS, resuspended in cold RLN-Igepal (50 mM Tris pH7.5, 140 mM NaCl, 1.5 mM MgCl_2_, 1 mM DTT, 0.4% v/v Igepal CA-630) for 5 min at a ratio of 4 million cells per ml and washed with cold RLN (50 mM Tris pH 7.5, 140 mM NaCl, 1.5 mM MgCl_2_, 1 mM DTT) to obtain purified nuclei. RNA was then extracted (4 million nuclei per column) using the RNeasy mini kit (Qiagen), treated with DNase for 1 h (Ambion), assessed for nuclear enrichment of intronic sequences by qRT–PCR (Supplementary Table 3) and then subjected to ribosomal RNA depletion using the RiboZero-gold kit (Illumina). First strand cDNA was prepared using Superscript III with random hexamer primers (Life Technologies) plus 240 ng Actinomycin C (Sigma-Aldrich) and purified from excess nucleotides using 1.8 × Ampure XT bead purification (Beckman-Coulter). The second cDNA strand was differentially marked by the incorporation of dUTP. Double-stranded cDNA was purified using QIAquick columns (QIAgen) and sheared to an average size of 300 bp (Covaris M220 sonicator, peak incidence power—50, Duty factor 10%, cycles per burst—200, treatment time—65 s). DNA ends were repaired using the NEBnext end repair module (NEB) and an adenine nucleotide overhang was introduced using Klenow exo- (NEB) in the presence of dATP, allowing ligation of Illumina Truseq adapters (NEB quick ligation kit). DNA was double-side size selected with 0.35 × –1 × Ampure XT bead selection to retain 250–700 bp fragments and the second strand of the cDNA was then degraded by incubation with UDG (NEB). This DNA was amplified with Illumina primers for 11 cycles and libraries sequenced either on a Hiseq 2000 (Illumina) using 100 × 2 bp paired-end reads or on a Nextseq 500 (Illumina) using 75 × 2 bp paired-end or 150 single-end reads.

Reads were mapped to the mm10 mouse genome using bowtie2 (ref. 37), after masking nucleotides located on SNPs from the Cast genome (Sanger mouse genome project—release 1505). Reads falling on SNPs were then sorted to genome of origin using SNPsplit^39^ (Babraham Institute) and PCR duplicates removed using picard (Broad Institute). Read coverage tracks were made using bedtools. Read pairs were assigned to a specific Vκ segment if they were transcribed in the same direction and resided within a region of 500 bp upstream to 10,000 bp downstream of the annotated Vκ segment. Statistical analyses were performed using the R statistical software package. Statistically significant monoallelic expression in the Vκ region was calculated as having an FDR corrected binomial *P*<0.05 and at least twice as high expression on one allele as compared to the other.

Genome-wide monoallelic transcripts were analysed as previously described^23^. Briefly, biological replicates of pre-B-cell libraries were analysed with the edgeR statistical package, utilizing a negative binomial model. Transcripts from the B6 allele from each replicate were assigned to one group, while transcripts from the Cast allele were assigned to the second group. Genes were assigned as monoallelic when they had an FDR-corrected *P*<0.05 and at least twice as high expression on one allele as compared to the opposite allele.

### Kinetics of V segment activation

By tabulating the number of V segments in the Igκ locus that are activated in each pre-B-cell clone, it was possible to calculate the probability of V-segment activation for both the B6 and Cast alleles. In clones 4 and 8, the probability of a V segment getting activated was 0.25 on the Cast allele and 0.43 on the B6 allele, and these *P* values accurately predicted the distribution of monoallelic and biallelic activation using a simple stochastic model, assuming that only 60 out of a total of 96 functional V segments have the potential to get turned on in all pre-B-cell clones. In clones 3 and B52, these probabilities were higher, but the ratio of B6/Cast remained the same.

### ATAC-seq library preparation and analysis

ATAC-seq was performed as described^40^. Briefly, 50,000 cells were washed with PBS and nuclei extracted with lysis buffer (10 mM Tris–Cl pH 7.5, 10 mM NaCl, 3 mM MgCl, 0.1 (v/v) Igepal CA-630). Nuclei were pelleted and resuspended in Nextera TD 1 × buffer containing the Tn5 Nextera transposase (Illumina), incubated for 30 min at 37 °C with mild agitation and the DNA then purified using QIAquick minelute columns (Qiagen) and amplified by PCR using Nextera complimentary primers. Libraries were pair-end sequenced (Nextseq 500—Illumina) and mapped using bowtie2 (ref. 37) to the mm10 mouse genome, where nucleotides located on SNPs from the Cast genome were masked (Sanger mouse genome project—release 1505). Reads falling on SNPs were then sorted to genome of origin using SNPsplit^39^ (Babraham Institute) and PCR-duplicate reads were removed with Picard. Peaks of open chromatin were called from all of the mapped reads using Homer software^41^. Allelic reads within peaks were counted using HTseq-count^38^. Peaks were considered to be monoallelic when they had an FDR-corrected binomial *P* value <0.05 and at least twice as high read counts on one allele as compared to the other consistently on biological duplicate libraries. Statistical analyses were performed using the R statistical software package.

### Igκ rearrangement repertoire analysis

DNase-treated RNA was taken either from MACS-selected CD19+ bone marrow cells from a 12-week-old female *B6/Cast* mouse or from pre-B-cell clones after IL-7 was removed from the culture media for 48 h (for all clones) or for a time course of 12, 24, 36 and 48 h (clone 4). RNA was poly-A enriched using poly-dT beads (Life Technologies) in two selection cycles and RT then performed using AffinityScript QPCR cDNA Synthesis Kit (Agilent) with an RT primer specific for the Cκ region 5′-ATGCTGTAGGTGCTGTCTTT-3′. The residual RNA was degraded with 0.1 N NaOH, neutralized with 0.1 M acetic acid and the single strand cDNA then purified using Silane beads (Life Technologies). A 3TR3 adapter (5′-/Phos/ AGATCGGAAGAGCACACGTCTG/3SpC3/-3′) was ligated to the 3′ end by overnight incubation with T4 RNA ligase (NEB) at 22 °C, and the cDNA then purified from excess adapter with Silane beads and PCR amplified for 12 cycles using the reverse complement of the 3RT3 adapter as the forward primer and the upstream Cκ region as the reverse primer with the partial Truseq Illumina adapter added to the beginning (5′-TACACGACGCTCTTCCGATCT-ACTGGATGGTGGGAAGATGGAT-3′). The PCR product was cleaned with 0.7 × ampure XT beads, amplified with indexed universal Illumina adapter primers for an additional seven cycles to obtain ∼550 bp libraries, which were sequenced (Miseq, 250 × 2 or 150 × 2 bp paired end). The resulting sequences were quality trimmed and aligned to a hybrid B6/Cast genome assembly using bowtie2 (ref. 37), with Cast polymorphic sites substituted based on the Sanger Mouse Genome Project database (release 1505). Reads over each Vκ segment were counted (HTseq-count) and normalized to the total mapped rearranged fragment number to allow comparison of the Vκ repertoire contribution between different libraries.

### Data availability

Sequence data that support the findings of this study have been deposited in the Gene Expression Omnibus (GEO) with the primary accession code GSE97148. Other data that support the findings of this study are available from the corresponding author upon request.

## Additional information

**How to cite this article:** Levin-Klein, R. *et al*. Clonally stable Vκ allelic choice instructs Igκ repertoire. *Nat. Commun.* **8,** 15575 doi: 10.1038/ncomms15575 (2017).

**Publisher's note:** Springer Nature remains neutral with regard to jurisdictional claims in published maps and institutional affiliations.

## Supplementary Material

Supplementary Information Supplementary Figures and Supplementary Tables
