# Supplementary material for: Clonally stable Vκ allelic choice instructs Igκ repertoire
Source: Nat Commun. 2017 May 30;8:15575. doi: 10.1038/ncomms15575 (PMC5459994; doi:10.1038/ncomms15575)
Supplement: Supplementary Information — Supplementary Figures and Supplementary Tables [file ncomms15575-s1.pdf]

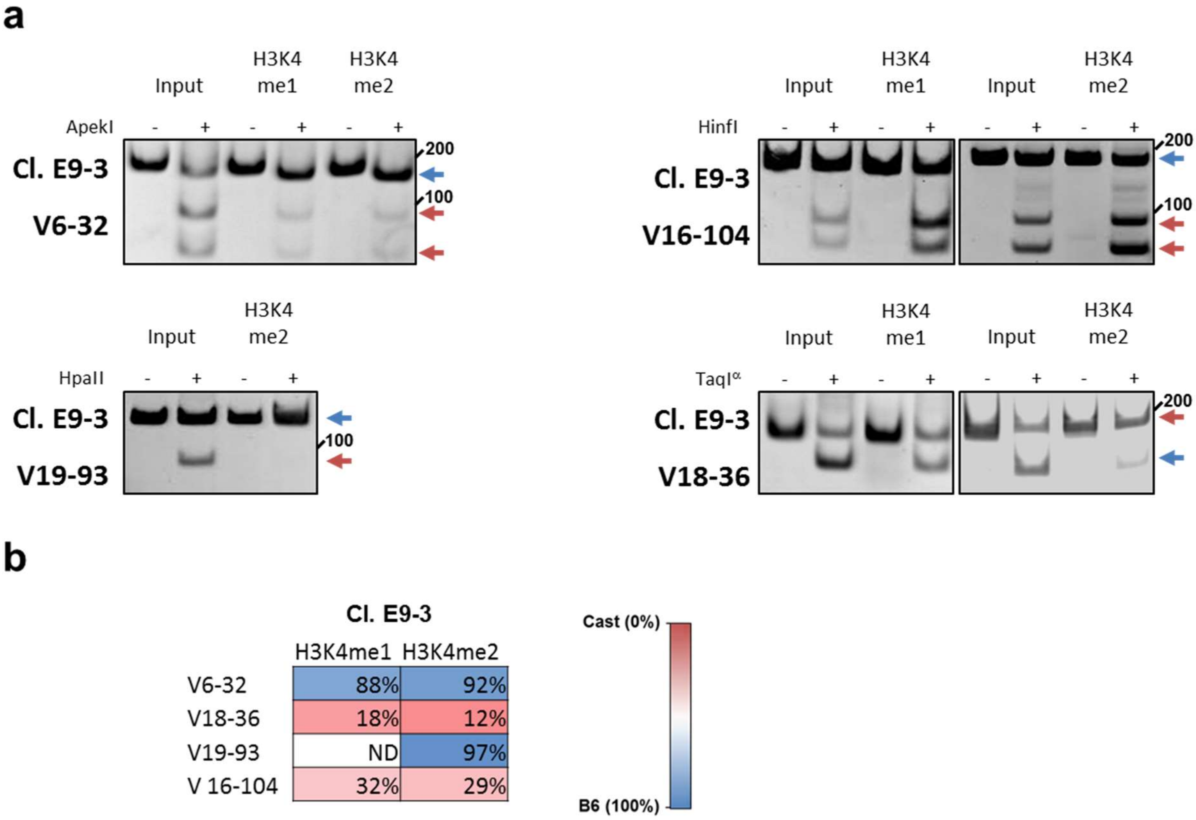

Supplementary Figure 1

(a) Restriction analysis gels from 4 different Vκ segments performed on H3K4me1 and H3K4me2 ChIP-enriched DNA from clone E9-3. Expected positions of Cast and B6 alleles following restriction is marked with red and blue arrows, respectively.

(b) Percent of the B6 allele within the ChIP bound fraction in clone E9-3, as quantified from the fraction of the PCR product cut in comparison to input, where the two alleles are present in equal proportions. Red to blue heatmap indicates Cast to B6 levels. Quantifications were done using EZquant software. ND – not determined.

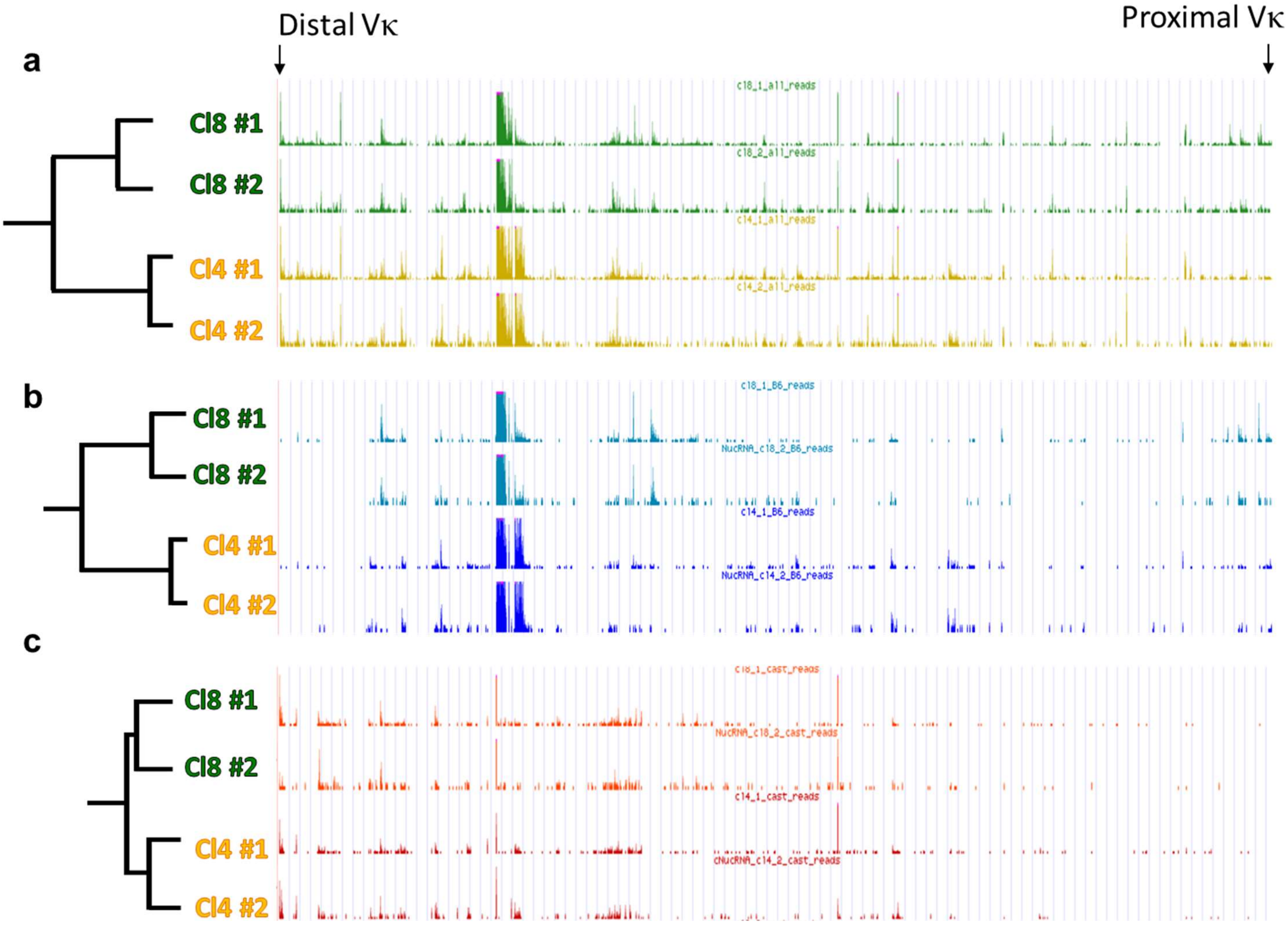

Supplementary Figure 2

Hierarchical clustering and Nuclear RNA profiles over the entire Vκ region of biological duplicates of clone 4 and clone 8.

(a) Total transcripts, **(b)** B6 transcripts, **(c)** Cast transcripts.

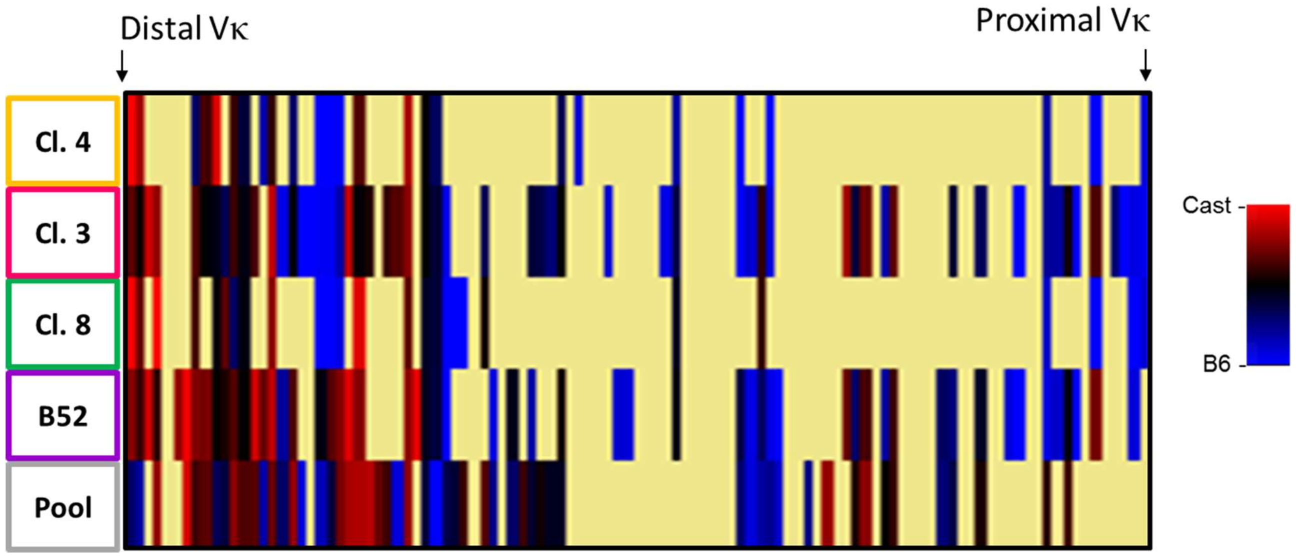

Supplementary Figure 3

Heatmap representing allelic expression of Vκ segments in 4 different pre-B cell clones and ex-vivo B6/Cast bone marrow pre-B cells (pool) as seen from nuclear RNA sequencing. Biological replicates were combined. Red to blue heatmap indicates linear Cast to B6 levels on a scale from 0 (100% Cast) to 1 (100% B6). Yellow indicates that the Vκ segment was expressed at low levels (<10 reads).

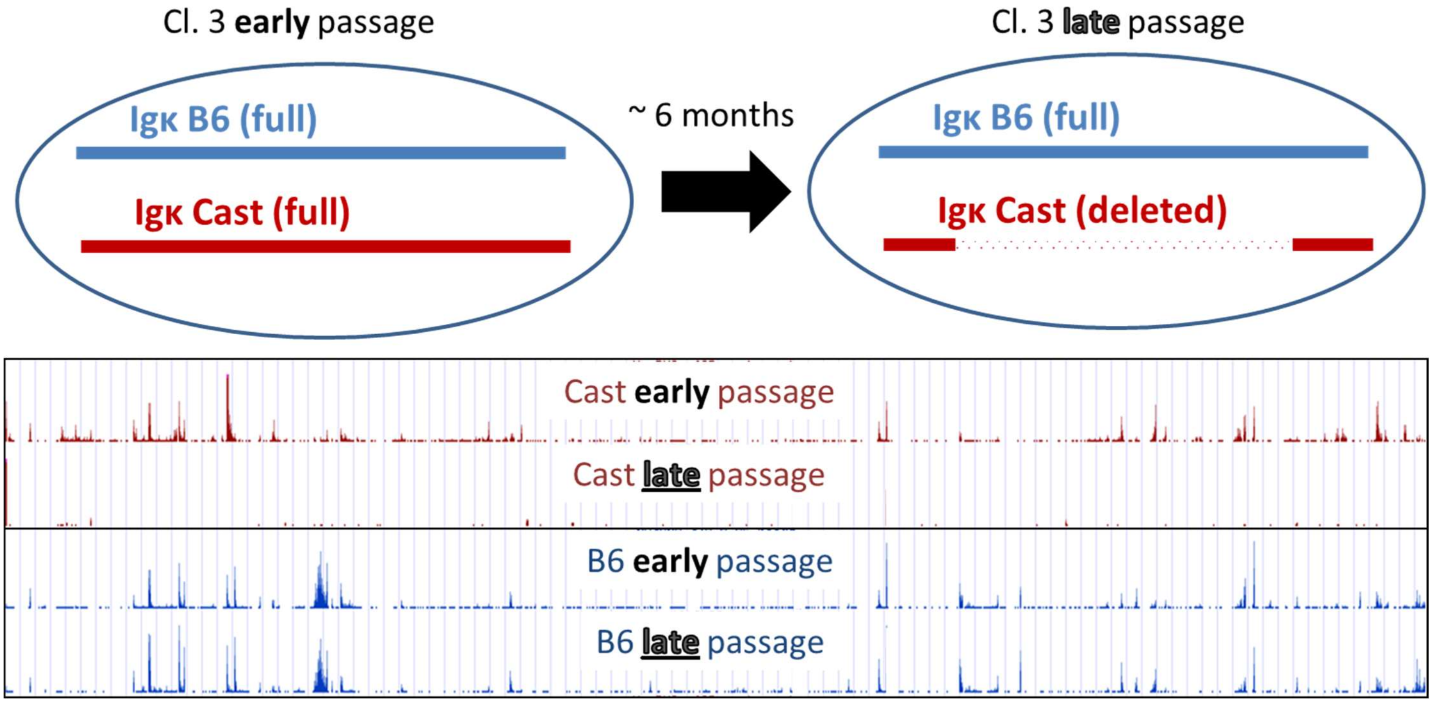

Supplementary Figure 4

Allelic nuclear RNA profile over Vκ region of early and late passage cells from clone 3. In the late passage, almost the entire Vκ region is deleted from the Cast allele due to a nonproductive rearrangement with the most distal Vκ segment.

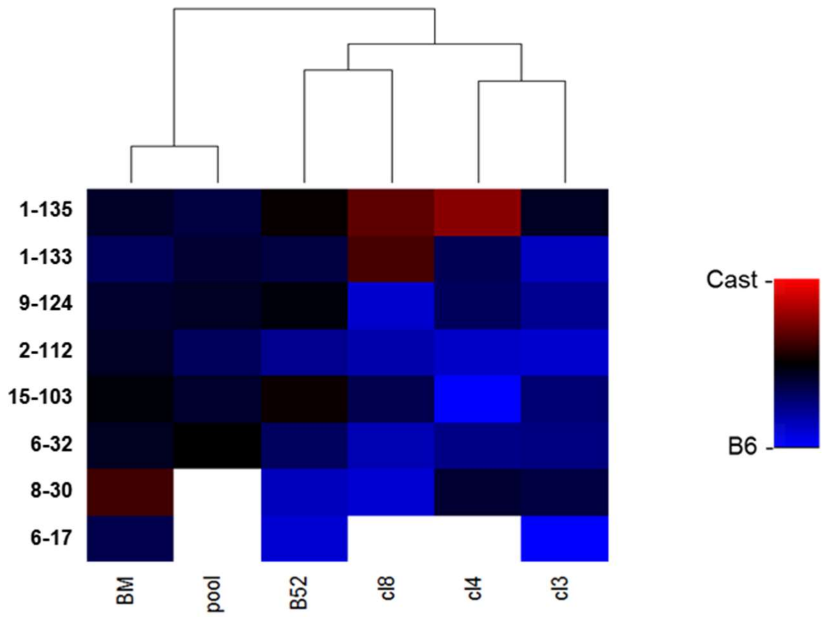

Supplementary Figure 5

Heatmap of allelic ratio of ATAC-seq peaks within Vκ promoters. Profiles derived from pre-B cell clones (B52, cl8, cl4, cl3), cultured bone marrow-derived pre-B cell pools (pool) and ex-vivo sorted pre-B cells (BM). Replicates were combined. Red to blue heatmap indicates linear Cast to B6 levels on a scale from 0 (100% Cast) to 1 (100% B6). White indicates that the peak had less than 10 reads. Peaks in intergenic regions of the Vκ locus were not included in this analysis.

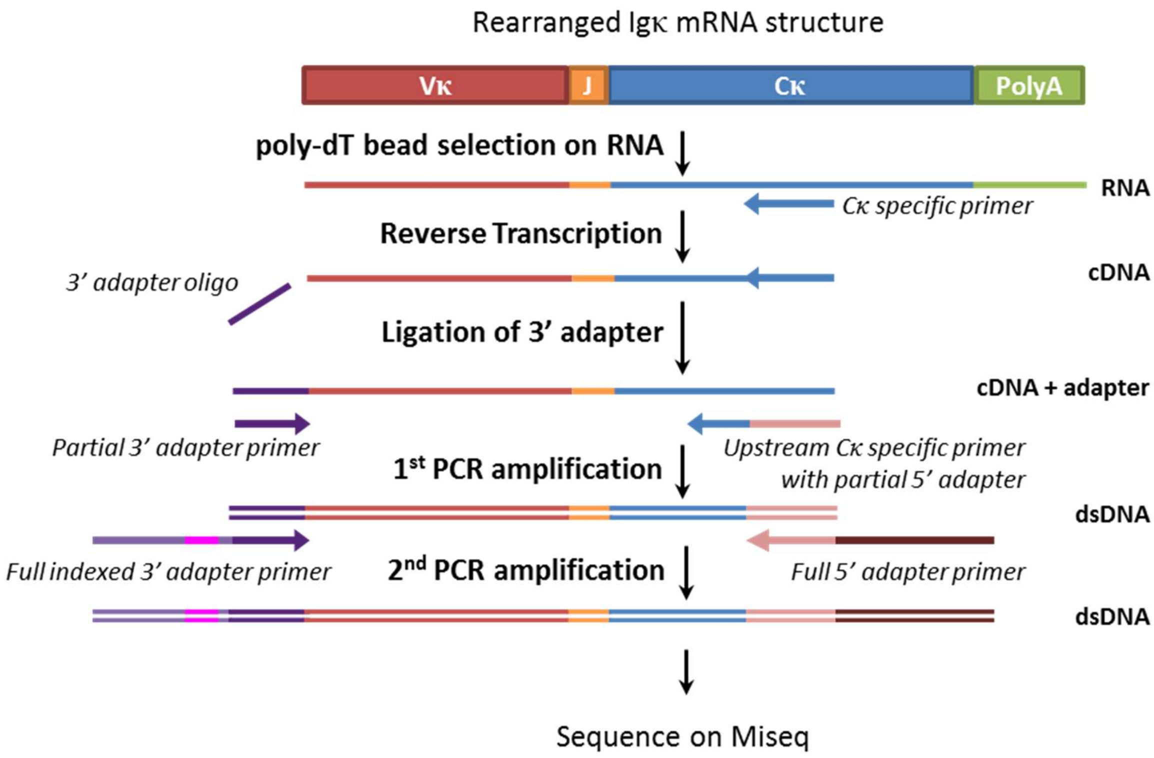

Supplementary Figure 6

Schematic overview of rearranged Igκ sequencing library preparation method.

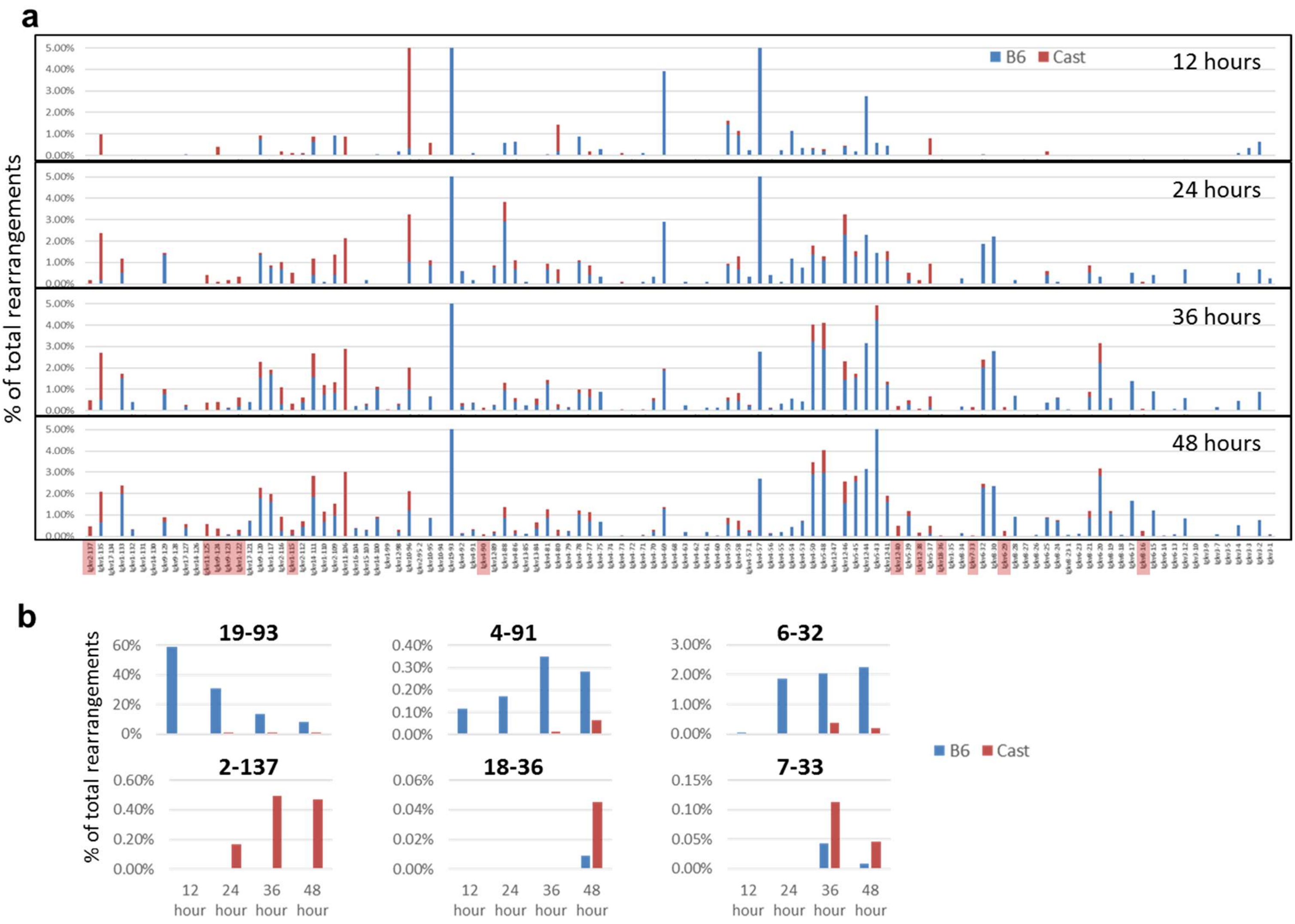

Supplementary Figure 7

(a) Allelic rearrangement repertoire of pre-B cell clone 4 following 12-48 hours of IL-7 withdrawal. As the cells remain without IL-7 signaling for more extended periods of time, the rearrangement repertoire becomes more varied, including a larger number of V segments. Additionally, more rearrangements are seen on the secondary allele (Cast in this case) at later time points. Many of the rearrangements on the secondary allele are unique (V segments name marked in red on X axis label). **(b)** Allelic rearrangement of selected number of V segments in clone 4, whose allelic accessibility pattern is known (see figures 1e and 2b). V segments accessible on the primary (B6) allele tend to rearrange on this allele already at early time points (top of panel). V segments, accessible on the secondary (Cast) allele, rearrange mostly on this secondary allele, and only at later time points (bottom of panel).

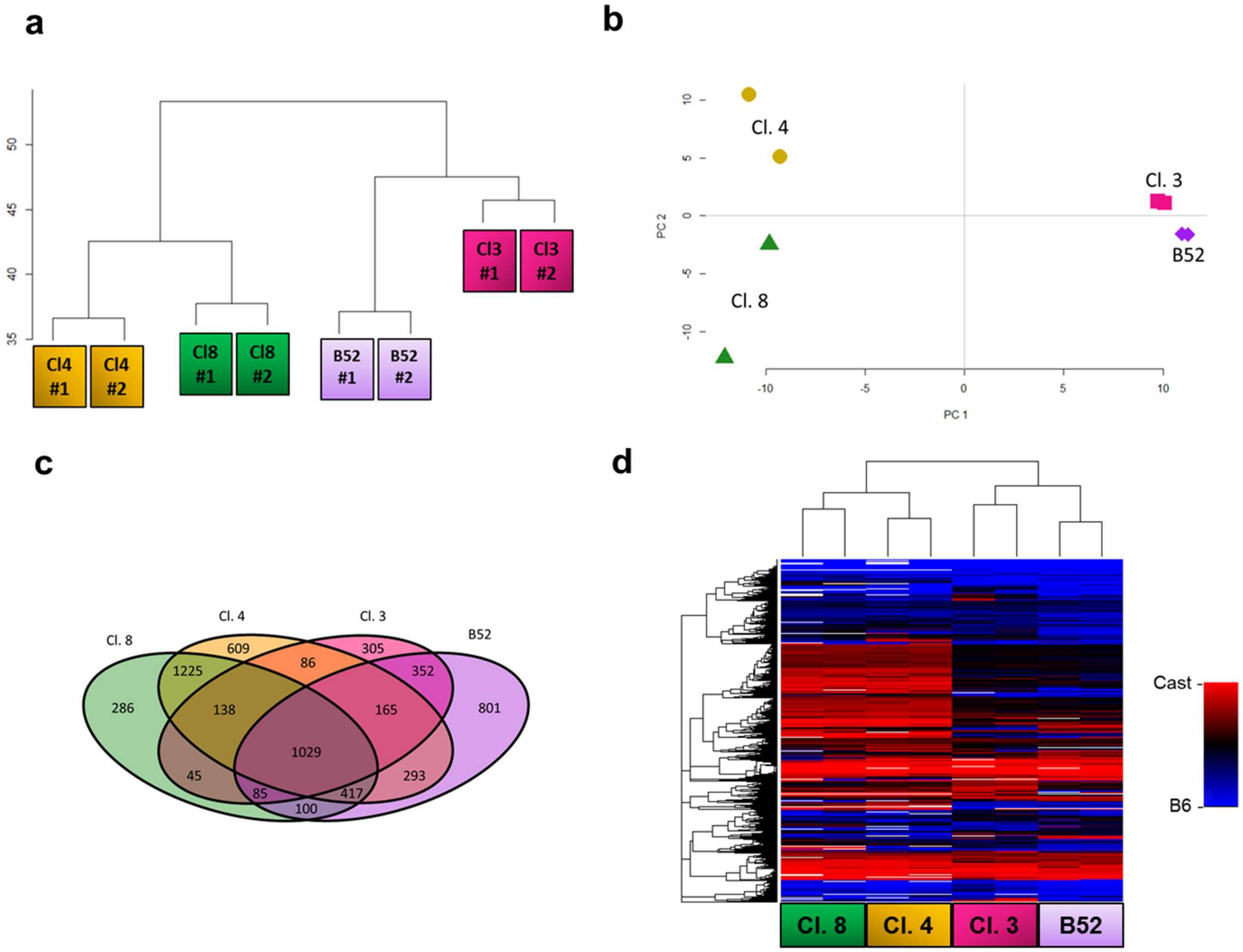

Supplementary Figure 8

Hierarchical clustering **(a)** of B6/Cast nuclear RNA ratios and principal component analysis **(b)** for all genes in pre-B cell clones. **(c)** Venn diagram of the unique and commonly monoallelic genes in 4 different pre-B cell clones. **(d)** Heatmap of nuclear RNA allelic ratios for all genes that are monoallelic in at least one pre-B cell clone. Red to blue heatmap indicates linear Cast to B6 levels on a scale from 0 (100% Cast) to 1 (100% B6). White indicates low expression (< 10 reads).

Supplementary Table 1. Primers used for Igκ V segment restriction analysis

Primer name	Primer sequence	Product size	Restriction Enzyme specificity (sizes of products)
ChIP4-91B6_L	AGCCAGGATTCTCCCCTAA	184bp	ApeKI cuts Cast (132,52)
ChIP4-91B6_R	TAGCACGGTGTGCGTGGTAT		
ChIP4-91cast_L	AGCCAGGATTCCCTCCTAAA		
ChIP4-91cast_R	TAGCACGGTGTGAGTGGTAA		
ChIP6-32L	AGTCCCTGATCGCTTCACTG	175bp	Cast cut by ApeKI (96,79)
ChIP6-32R	TAGGACAGGCAGCTGTGAGA		
ChIP7-33_L	GGCCAGTGAGAGCCTTTATTC	157bp	B6 cut by ApaLI (35,122)
ChIP7-33_R	ATCTGTCCCAGATCCACTGC		Cast cut by HinfI (108,49)
ChIP15-103L	AGTGGCAGTGGATCTGGAAC	169bp	B6 cut by HincII(79,90)
ChIP15-103_cast_R	AGCTGAGGCAGCACAGASTT		
ChIP15-103_B6_R	AGCTGAGGCAGCACAGACTT		
ChIP19-93L	CCTGGAGCCTGAAGATATTGC	128bp	Cast cut by HpaII(87,41)
ChIP19-93_B6_R	GAATTTGTGGGCAACCTAGC		
ChIP19-93_cast_R	GAATTTGGGGGCAACCTAGC		
ChIP18-36_B6_L	ACTGTGGACCACGGGTAAGA	192bp	B6 cut by TaqI$^{\alpha}$ (140,52)
ChIP18-36R	TGACAGCTGAGACTGAAAAGG		
ChIP18-36_cast_L	ACTGTGGACGGGTAAGAGGA		
ChIP16-104L	GGTTCAGTGGCAGTGGATCT	173bp	Cast cut by HinfI (96,77)
ChIP16-104R	AATGGCTAGCCCAACATCTG		

Supplementary Table 2. Primers used for Igκ V amplicon sequencing

Primer name	Primer sequence
mi-V2-137F	GAGTTCTACAGTCCGACGATCGAGGTGCCTAGCTGAGTTCC
mi-V2-137R	CCTTGGCACCCGAGAATTCCAGAGGTTTTGTTCAGGGCTGT
mi-v1-135F	GAGTTCTACAGTCCGACGATCTGCCCAGTTCCTGTTTCTGT
mi-v1-135R	CCTTGGCACCCGAGAATTCCACCAGGCAAGGAAGTTTTTGTT
mi-v17-121F	GAGTTCTACAGTCCGACGATCCCTCAGTCTTCTTCTCCTCTGTG
mi-v17-121R	CCTTGGCACCCGAGAATTCCATGACAGCATCTGCAGGTTTC
mi-v1-117F	GAGTTCTACAGTCCGACGATCGTTGGTGCTGATGTTCTGGA
mi-v1-117R B6	CCTTGGCACCCGAGAATTCCATTTAGCAGCTGTGATACCCAAA
mi-v1-117R cast	CCTTGGCACCCGAGAATTCCATTTAGCAGCTGGGATACCCAAA
mi-V1-110F	GAGTTCTACAGTCCGACGATCGTTGGTGCTGATGTTCTGGA
mi-V1-110R	CCTTGGCACCCGAGAATTCCATAGCAGCTGGGATACCCAAA
mi-v19-93F	GAGTTCTACAGTCCGACGATCATTCAGTTCCTGGGGCTCTT
mi-v19-93R	CCTTGGCACCCGAGAATTCCAGGGCAACCTAGCCTCTCACT
mi-v1-88F	GAGTTCTACAGTCCGACGATCTGGTGCTTCTGTTGTTCTGG
mi-v1-88R	CCTTGGCACCCGAGAATTCCAGGCCACTCTAAGCAAGGAAGT
mi-v12-46F	GAGTTCTACAGTCCGACGATCTGCTGCTGCTGTGGCTTA
mi-v12-46R	CCTTGGCACCCGAGAATTCCATCCCTGCATGGTTTATGTCA
mi-v8-21F	GAGTTCTACAGTCCGACGATCTTGCTGCTGCTATGGGTATCT
mi-v8-21R	CCTTGGCACCCGAGAATTCCACAGGCAGCTGGTGAAACTCT
mi-v3-2F	GAGTTCTACAGTCCGACGATCAGACACACTCCTGCTATGGG
mi-v3-2R	CCTTGGCACCCGAGAATTCCACCAGGAGGTTTTGTTCAGC

Supplementary Table 3. Primers used to assess nuclear RNA enrichment by qPCR

	Primer name	Sequence
Cytoplasmic	28S rRNA F	GCGACCTCAGATCAGACGTGG
	28S rRNA R	CTGTTCACTCGCCGTTACTGAG
	Gapdh exon3 a f	GGCCAAAAGGGTCATCATCTC
	Gapdh exon3 a r	CCTTCCACAATGCCAAAGTT
Nuclear	45S rRNA F	CTCTTGTTCTGTGTCTGCC
	45S rRNA R	GCCCGCTGGCAGAACGAGAAG
	Gapdh intron 2-3 F	GGAAAGAAACCTCCACTTTATAACC
	Gapdh intron 2-3 R	ATAGACTGTTCCCACCCTAGAAAGT
	Myc ex1	GCTGTTTGAAGGCTGGATTTCCT
	Myc int1	CGCTACATTCAAGACGCAGAAAGA
